# Common Diseases Presenting in an Uncommon Way: Metastatic Small-Cell Lung Carcinoma Mimicking Merkel Cell Carcinoma

**DOI:** 10.7759/cureus.84968

**Published:** 2025-05-28

**Authors:** Alice Boican

**Affiliations:** 1 Internal Medicine, HCA Healthcare / University of South Florida (USF) Morsani College of Medicine Graduate Medical Education, HCA Florida Sarasota Doctors Hospital, Sarasota, USA

**Keywords:** merkel cell cancer, metastatic basal cell carcinoma, monoclonal antibody therapy, rare cancers, small-cell lung carcinoma

## Abstract

A 65-year-old Caucasian woman presented with a rapidly enlarging, painless forehead lesion initially presumed to be Merkel cell carcinoma (MCC). Originating as a 3 mm pink papule, the lesion expanded to over 12 mm within six months. Subsequent histopathologic and radiologic evaluation revealed it to be the initial manifestation of metastatic small-cell lung carcinoma (SCLC). Despite undergoing surgical resection under the presumption of localized MCC, advanced imaging later identified a primary pulmonary neoplasm with diffuse metastatic spread. Histologically, both MCC and SCLC are characterized by poorly differentiated neuroendocrine morphology, complicating clinical distinction. Cutaneous metastases from SCLC, although rare, can deceptively mimic primary neuroendocrine skin neoplasms, as exemplified in this case. This diagnostic pitfall underscores the imperative for comprehensive staging prior to initiating definitive therapy, as incomplete evaluation may precipitate unnecessary surgical intervention and delay optimal systemic treatment.

## Introduction

Small-cell lung carcinoma (SCLC) is a malignant and rapidly proliferating form of lung cancer that originates from neuroendocrine cells in the bronchial epithelium. Although it contributes to approximately 13% of all lung cancer cases, its aggressive nature contributes disproportionately to lung cancer mortality [1]. SCLC is intimately associated with chronic tobacco exposure, with a strong epidemiological correlation indicating that nearly all patients have a significant smoking history or cigarette smoke exposure. The presence of potent carcinogens in tobacco smoke, particularly nitrosamines and polycyclic aromatic hydrocarbons, induces DNA damage and promotes genomic instability [2]. These carcinogens interfere with normal cellular processes by disrupting tumor suppressor genes, such as TP53 and RB1, and impairing apoptosis, thereby promoting unrestricted cellular proliferation.

Epidemiological models estimate over 226,000 new lung cancer cases in the United States in 2025, with a significant fraction being SCLC [1]. There is also a dose-response relationship between smoking intensity and duration and the risk of developing SCLC, highlighting the importance of cumulative exposure. This means that the longer and more frequently a person smokes, the higher their risk of developing SCLC [2].

Clinically, SCLC is characterized by a high mitotic index, rapid tumor doubling time, and a strong propensity for early hematogenous and lymphatic dissemination. Unfortunately, most patients are diagnosed at an advanced stage, often with metastases to the liver, brain, bone, or, rarely, the skin. While SCLC is fundamentally a thoracic disease, cutaneous metastases, although uncommon, may occur and are generally considered a poor prognostic sign, indicating widespread systemic disease [1]. These skin lesions are typically nodular and non-painful and may appear similar to primary skin cancers, making diagnosis complex without histological analysis.

In contrast, Merkel cell carcinoma (MCC) is a primary neuroendocrine carcinoma of the skin, arising from mechanoreceptor Merkel cells or a similar progenitor cell. Its incidence has been steadily increasing, particularly among the elderly, immunocompromised, or organ transplant recipients [3]. By 2025, projections suggest that approximately 3,250 new MCC cases will occur in the United States [4]. MCC typically manifests as a firm, painless, violaceous, or red nodule located on sun-exposed skin, such as the face, neck, or upper extremities. These lesions can be clinically mistaken for cysts, lipomas, basal cell, or squamous cell carcinomas, highlighting the importance of histopathological and immunohistochemical evaluation for accurate diagnosis.

## Case presentation

A 65-year-old Caucasian woman with a long-standing history of heavy tobacco use presented to her primary care physician after she noticed a small pink bump, approximately 3 mm in size, and over the next six months, it gradually grew into a firm, red, subcutaneous mass measuring nearly 2 cm. She reported no pain, itching, bleeding, or ulceration at that time. The lesion was firm, non-mobile, and located just above the left eyebrow, with some overlying redness without ulceration, bruising, or attachment to the bone underneath. Her left supraclavicular lymph nodes were slightly enlarged but soft, mobile, and non-tender. Her lungs were clear on auscultation, with normal breath sounds throughout, though slightly diminished over the left upper chest. She denied recent rapid weight loss. She was referred by her primary care physician to a plastic surgeon who immediately performed resection.

**Figure 1:**
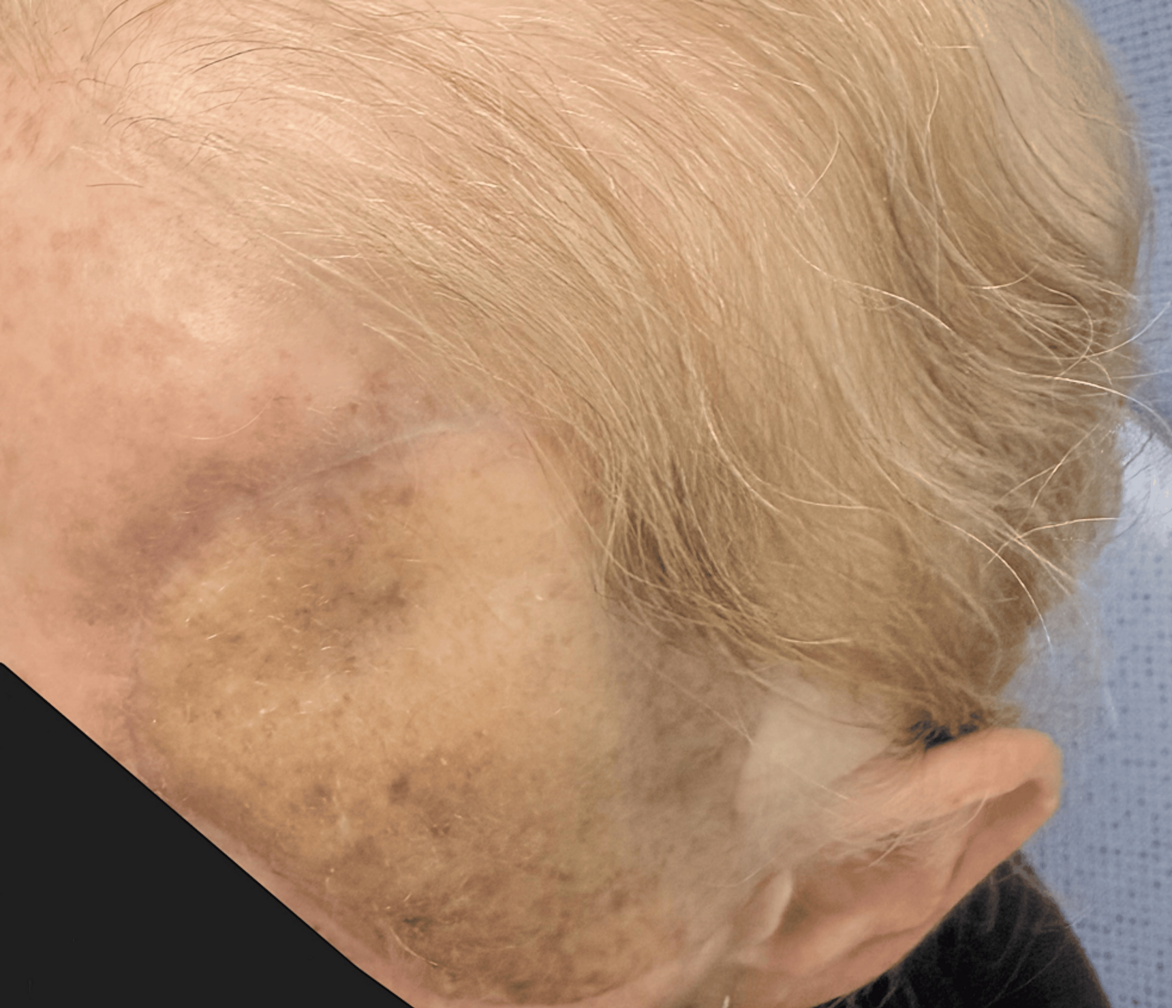
Image depicting the patient following surgical resection and skin graft. Unfortunately, the patient presented to oncology after invasive resection.

She presented to oncology status post-resection and skin graft reconstruction on the left side of her forehead (Figure 1); unfortunately, no prior images were provided by her plastic surgeon. Given the lesion's concerning histologic features and potential for systemic spread, a full-body PET-CT scan was performed. This revealed multiple areas of abnormal metabolic activity, notably in the left frontotemporal bone, supraclavicular lymph nodes, mediastinal nodes, and multiple sites throughout the skeletal system, consistent with widespread metastatic disease. Fortunately, an MRI of the brain was negative, with no signs of intracranial involvement.

As part of the thoracic workup, a bronchoscopy was performed to investigate subtle radiologic abnormalities noted in the left mainstem bronchus. A small endobronchial lesion was visualized and biopsied. Histopathological analysis confirmed a high-grade neuroendocrine carcinoma. To further clarify the tumor's origin, immunohistochemical staining was conducted. The tumor cells were strongly positive for markers associated with neuroendocrine differentiation (synaptophysin, chromogranin, CD56, and CAM 5.2) and, most notably, TTF-1, a nuclear marker highly suggestive of pulmonary origin. In addition, the Ki-67 proliferation index was markedly elevated, indicating a very aggressive tumor biology. The tumor was negative for CK20, CK7, CK5, and CD45, helping to rule out MCC and other epithelial or hematolymphoid malignancies. These findings, summarized in Table 1, confirmed the diagnosis of extensive-stage SCLC.

**Table 1:** Immunohistochemical markers of neoplastic skin lesions and their pathological implications. SCLC: small-cell lung carcinoma; MCC: Merkel cell carcinoma

Marker	Result	Reference Expression	Interpretation/Utility
CAM 5.2	Positive	Positive in epithelial tumors	Confirms epithelial origin
Synaptophysin	Positive	Positive in neuroendocrine tumors	Supports neuroendocrine differentiation
Chromogranin	Positive	Positive in neuroendocrine tumors	Confirms neuroendocrine lineage
CD56	Positive	Positive in SCLC and MCC	Nonspecific marker; indicates neuroendocrine origin
TTF-1	Positive	Typically positive in SCLC; negative in MCC	Strongly favors pulmonary (SCLC) origin
Ki-67	High (>80%)	>80% in SCLC; lower in MCC (typically <55%)	Indicates a high proliferative index consistent with SCLC
CK20	Negative	Positive in MCC (dot-like)	Negativity rules against MCC
CK7	Negative	Variable; less relevant for distinction	Nonspecific
CK5	Negative	Basal keratin; not expected in SCLC	Rules out squamous/basal subtype
CD45	Negative	Marker for hematopoietic cells	Rules out lymphoma/leukemia

Once the diagnosis was established, the patient was started on systemic therapy with a combination of carboplatin and etoposide, standard chemotherapy agents used in SCLC, along with atezolizumab, an immune checkpoint inhibitor that has been shown to improve outcomes in extensive-stage disease [5]. Unfortunately, there was a delay in initiating treatment due to the complexity of the diagnostic process. Earlier recognition of the lesion's metastatic potential and prompt coordination between specialties might have expedited the diagnostic timeline, allowing for quicker initiation of treatment.

## Discussion

This case highlights the real-world challenge of distinguishing metastatic SCLC from primary cutaneous neuroendocrine tumors like MCC. Both cancers share similar histologic features, including small round blue cell morphology, which can make differentiation difficult on microscopy alone [6]. This overlap becomes particularly relevant when SCLC presents with cutaneous lesions, as in our case. For clinicians, the risk lies in mistaking metastatic disease for a primary skin cancer, leading to localized treatments that may not address the underlying systemic pathology [7].

Our patient's lesion was initially suspected to be MCC based on clinical presentation: location, appearance, and rapid progression. However, immunohistochemical analysis revealed a different story. The tumor was positive for TTF-1 and negative for CK20, findings more consistent with SCLC. This aligns with prior studies showing that while both MCC and SCLC express neuroendocrine markers like synaptophysin, chromogranin, and CD56, TTF-1 and CK20 are more reliable discriminators [8]. It is important to note that the interpretation of these stains can be tricky; recent reports have described aberrant expression patterns, such as BCOR positivity in MCC, which can create additional diagnostic confusion [6]. This reinforces the importance of using a comprehensive panel of markers rather than relying on single immunostains.

Imaging plays an equally important role. Although cutaneous metastases are relatively uncommon overall, lung cancer, particularly SCLC, is among the more likely primary sources when they do occur. Some estimates suggest that SCLC accounts for up to 24% of all cases of cutaneous metastasis [9]. PET-CT and brain MRI were instrumental in our case, revealing the extent of systemic involvement and guiding oncologic management. Had imaging been delayed, the patient could have undergone more extensive surgery with no benefit, a scenario that unfortunately occurs in similar cases [10].

Treatment approaches between SCLC and MCC differ significantly. MCC can be treated surgically when localized and often includes lymph node evaluation and radiotherapy. Immunotherapy has gained ground for advanced MCC, especially with PD-1/PD-L1 inhibitors [11]. In contrast, metastatic SCLC requires systemic treatment from the outset. Chemotherapy combined with immunotherapy remains the standard, with surgery rarely playing a role [12]. This makes an accurate diagnosis upfront critical, as it directly impacts treatment planning.

This case underscores the need for a thorough diagnostic workup, including biopsy, immunohistochemistry, and full-body imaging, especially in high-risk patients like chronic smokers. A rapidly growing skin nodule in this context should not be assumed to be a primary skin cancer without further evaluation. Collaboration across dermatology, oncology, pathology, and radiology teams is essential. Had we acted on appearance alone, this patient might have received inappropriate local therapy, delaying the systemic treatment she actually needed. The lesson is clear: when neuroendocrine tumors appear in the skin, always think beyond the surface.

## Conclusions

This case highlights the diagnostic complexity that can arise when common malignancies present in uncommon ways. A seemingly benign cutaneous lesion in a patient with a long history of tobacco use was initially presumed to be a primary skin cancer but ultimately represented a cutaneous metastasis from an underlying SCLC. This underscores the critical importance of considering a wide differential diagnosis in high-risk patients, particularly when lesions display rapid progression or atypical features. Clinical judgment alone may not be sufficient to distinguish between primary cutaneous neuroendocrine tumors and metastatic disease, especially when histopathologic characteristics overlap. Immunohistochemistry and comprehensive imaging played a pivotal role in establishing the correct diagnosis in this case and should be pursued early in the diagnostic process when there is any uncertainty.

Moreover, the delay in initiating systemic therapy due to misclassification highlights the real-world consequences of diagnostic delays in aggressive malignancies. This case reinforces the need for a multidisciplinary approach and timely oncologic referral in similar presentations. Recognizing subtle but significant clinical clues, integrating histologic findings with systemic assessment, and avoiding assumptions based on lesion appearance alone are all essential to delivering accurate, timely, and effective care in patients with potentially life-threatening diseases.
